# In Vitro Evaluation of Novel Hybrid Cooperative Complexes in a Wound Healing Model: A Step Toward Improved Bioreparation

**DOI:** 10.3390/ijms20194727

**Published:** 2019-09-24

**Authors:** Antonella D’Agostino, Rosa Maritato, Annalisa La Gatta, Alessandra Fusco, Sabrina Reale, Antonietta Stellavato, Anna Virginia Adriana Pirozzi, Mario De Rosa, Giovanna Donnarumma, Chiara Schiraldi

**Affiliations:** Department of Experimental Medicine, School of Medicine, University of Campania “Luigi Vanvitelli,” Via L. De Crecchio 7, 80138 Naples, Italy; rosa.maritato@unicampania.it (R.M.); annalisa.lagatta@unicampania.it (A.L.G.); alessandra.fusco@unicampania.it (A.F.); sabrinareale88@gmail.com (S.R.); antonietta.stellavato@unicampania.it (A.S.); annavirginiaadriana.pirozzi@unicampania.it (A.V.A.P.); mario.derosa@unicampania.it (M.D.R.); giovanna.donnarumma@unicampania.it (G.D.)

**Keywords:** wound healing, inflammation, hyaluronic acid, biorepair

## Abstract

The effectiveness of hyaluronic acid (HA), also called as hyaluronan, and its formulations on tissue regeneration and epidermal disease is well-documented. High-molecular-weight hyaluronan (HHA) is an efficient space filler that maintains hydration, serves as a substrate for proteoglycan assembly, and is involved in wound healing. Recently, an innovative hybrid cooperative complex (HCC) of high- and low-molecular-weight hyaluronan was developed that is effective in wound healing and bioremodeling. The HCC proposed here consisted of a new formulation and contained 1.6 ± 0.1 kDa HHA and 250 ± 7 kDa LHA (low molecular weight hyaluronic acid). We investigated the performance of this HCC in a novel in vitro HaCaT (immortalized human keratinocytes)/HDF (human dermal fibroblast) co-culture model to assess its ability to repair skin tissue lesions. Compared to linear HA samples, HCC reduced the biomarkers of inflammation (Transforming Growth Factor-β (TGF-β), Tumor Necrosis Factor receptor-α (TNF-α), interleukin-6 (IL-6), and interleukin-8 (IL-8)), and accelerated the healing process. These data were confirmed by the modulation of metalloproteases (MMPs) and elastin, and were compatible with a prospectively reduced risk of scar formation. We also examined the expression of defensin-2, an antimicrobial peptide, in the presence of hyaluronan, showing a higher expression in the HCC-treated samples and suggesting a potential increase in antibacterial and immunomodulatory functions. Based on these in vitro data, the presence of HCC in creams or dressings would be expected to enhance the resolution of inflammation and accelerate the skin wound healing process.

## 1. Introduction

There is growing interest in unraveling the correlation between hyaluronan (HA) and inflammation in response to stress conditions or damaged tissue (i.e., wound healing (WH)). HA is widely applied as natural, modified, or auto-crosslinked molecules, or crosslinked with other molecules in different biomedical fields, including orthopedics, ophthalmology, dentistry, tissue repair, and cosmetics. The success of the medical applications of HA has led to several commercial products, a number of which have been evaluated in previous studies [1,2,3,4].

HA is one of the most frequently used formulations for wounds, burns, and ulcer treatments, which often consist of specific hydrogels, such as alginate, carboxymethylcellulose, or chitosan [5,6]. The use of creams (foams and ointments) for infectious wounds is usually based on natural or synthetic biocompatible macromolecules with a higher viscosity, and often contain antibiotics and/or antibacterial agents [7,8,9,10,11,12,13]. Regarding commercial formulations containing HA, numerous products are reported to reduce ulcerous areas, which relieves symptoms related to skin burns and quickly decreases the wound size in general [14].

Especially in the dermal regeneration field, lower-molecular-weight (50–300 kDa) HA is recognized as having a key role, as it has demonstrated higher skin penetration rates, in contrast to the scientific literature indicating the impermeability of high-molecular-weight HA (HHA; 1000–1400 kDa). In a recent study, we confirmed that epithelial cells treated with low-molecular-weight HA (LHA; 50–100 kDa) have higher *RHAMM* gene expression with respect to the control, supporting the prominent role of LHA in cellular migration [15].

Many formulations of HA have been commercialized as part of medical devices. They contain medium–low-molecular-weight hyaluronic acid (about 0.2% *w*/*w*) and are topical systems widely used to treat wounds and acute, or even chronic, conditions, such as leg ulcers. Despite the large number of medical devices and topical formulations supported by clinical studies, there is a further need for reliable in vitro models to better assess the potential functionality of novel formulations to target the specific lesions in relation to the patient’s pathophysiological status, age, and the nature of the wound (even chronic), which can also be infected.

The present study aimed to evaluate novel hybrid cooperative complexes (HCCs) obtained through NaHyCo® technology [16] and containing both high- and low-molecular-weight hyaluronic acid (HHA ≈ 1600 kDa and LHA ≈ 220 kDa) for biomedical applications, especially topical wound treatment. In this particular case, we used different molecular weights than the ones evaluated in previous research [15] in order to obtain more viscous and adhesive creams suitable for topical application, even on infectious wounds.

For this purpose, we attempted to mimic the infectious environment on human epidermal cells in vitro by applying both a mechanical scratch and an inflammatory insult in order to better simulate a chronic wound.

Specifically, interleukin (IL)-1β was added in the medium of in vitro cell cultures to mimic the in vivo inflammatory status. In fact, IL-1β is a particularly potent pyrogenic cytokine and a key modulator involved in the immune response [17,18,19,20,21]. Co-cultures of keratinocytes and fibroblasts were used, scratched or not, with and without interleukin (cytokine based). Time-lapse video microscopy was exploited as a preliminary screening to visualize and record the cell migration and scratch closure. To investigate the biological pathways involved in these repair mechanisms, we analyzed specific and well-known bioremodeling markers and inflammation-associated genes, such as metalloproteinases (MMPs) and elastin. Furthermore, the role of human β-defensin-2 (HBD2) was analyzed. HBD-2 is an antimicrobial peptide that is naturally produced by human skin that exhibits activity against Gram-positive and Gram-negative bacteria and fungi [22], whose expression is induced in cultured epithelial cells by pro-inflammatory cytokines, microorganisms, and other factors of inflammation. The presence of HBD-2 on the outer layers of the epidermis also gives it a role in innate immunity, and in the wound healing, not only for its antimicrobial activity, but also for its ability to exert chemotactic activity on other types of cells primarily implicated in tissue repair processes [23,24].

## 2. Results

### 2.1. Hydrodynamic and Rheological Characterization

**Figure 1:**
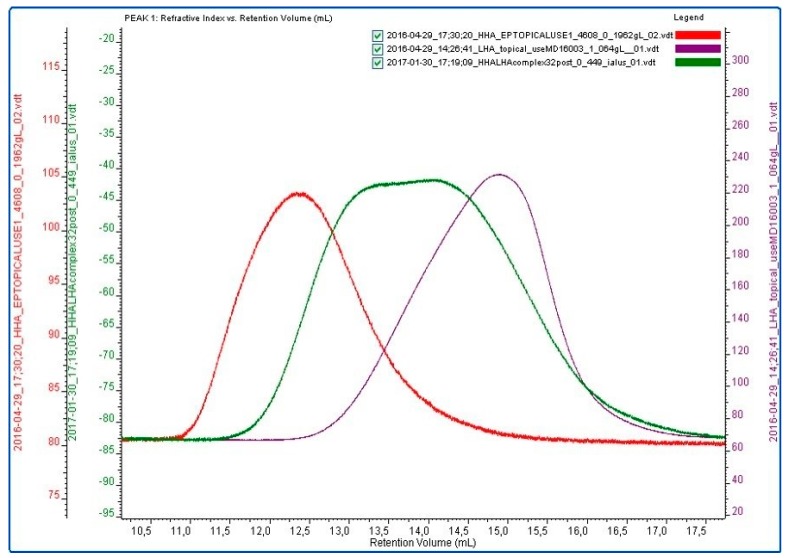
Refractive index (RI) measurements on Size Exclusion Chromatography -Triplo Detector Array (SEC-TDA) analyses. Overlay of HHA (red), LHA (violet), and HCC samples (green) after thermal treatment.

The chromatographic profiles of HHA, LHA, and HCC after thermal treatment are reported in Figure 1. The overlay supports HCC formation. The HHA, LHA, and HCC samples were diluted to the concentration used for the biological experiments and investigated for their dynamic viscosity (Table 1). The η_0_ values were in the range of 4–95 mPa∙s, which varied consistently with the biopolymer molecular weight [3]. According to the data in the literature, no dependence on the shear rate was found for the LHA sample’s viscosity [3]. As expected, both the HHA and HCC samples exhibited a viscosity that decreased with the shear rate.

### 2.2. Biological Response of HaCaT/HDF to HA-Based Treatments

#### 2.2.1. Elastin Expression

Elastin was examined using two different models: the first, using an immunofluorescence (IF assay), adding only the HA based gels (without cytokine stress) to the co-culture; and the second, using Western blotting on extracted proteins of a scratched confluent monolayer in the presence of the HA treatments.

**Figure 2:**
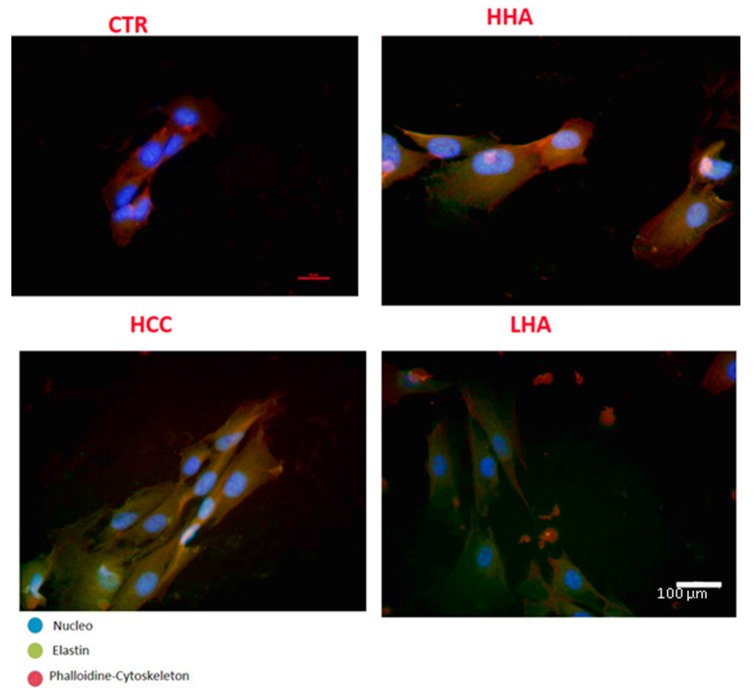
Expression of elastin in HaCaT/HDF co-cultures for a control and in the presence of HHA, LHA, and HCC. Images were taken after 24 h of treatment. The panels show triple immunofluorescence analysis for cytoskeleton (red), nucleus (blue), and elastin (green). In the presence of HCC, the expression of elastin was increased. Scale bar represents 100 μm.

**Figure 3:**
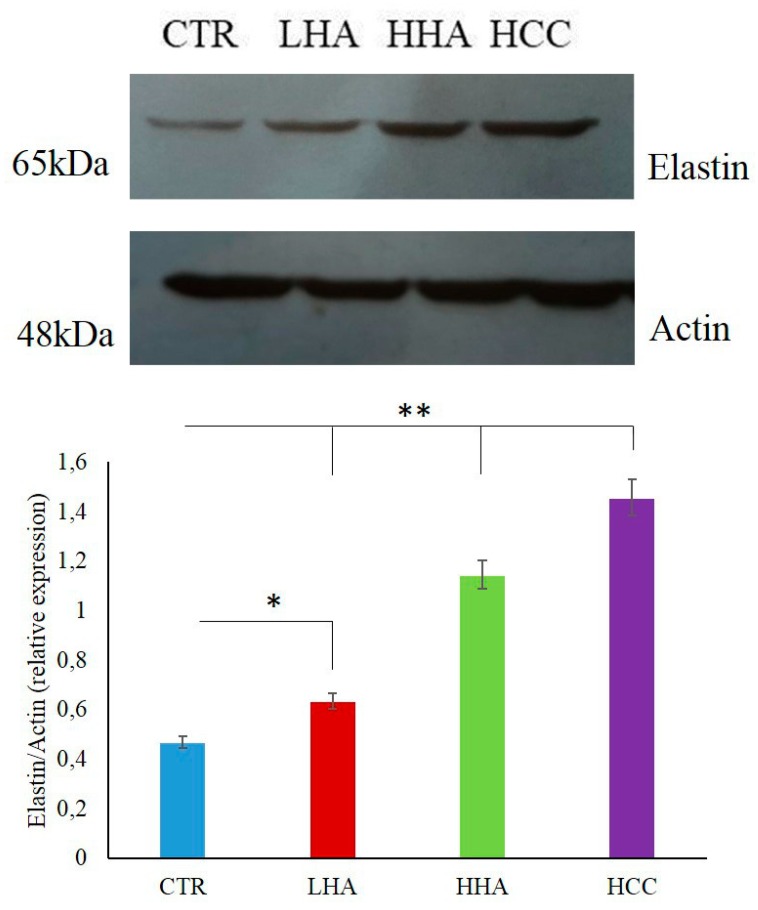
Effects of LHA, HHA, and HCC on elastin protein expressions in scratched HaCaT cells at 24 h in comparison with a control sample. Densitometry values are normalized with respect to actin. *p < 0.05 for LHA vs. ctr; **p < 0.01 for HHA and HCC vs. CTR.

When elastin expression was analyzed using immunofluorescence, all treatments improved protein expression with respect to the control. In particular, a slight increase was observed in the presence of HCC versus HHA and LHA (Figure 2). Western blotting (Figure 3) indicated that elastin protein was up-regulated by all treatments, but especially in the presence of HCC.

#### 2.2.2. HBD-2 Modulation

**Figure 4:**
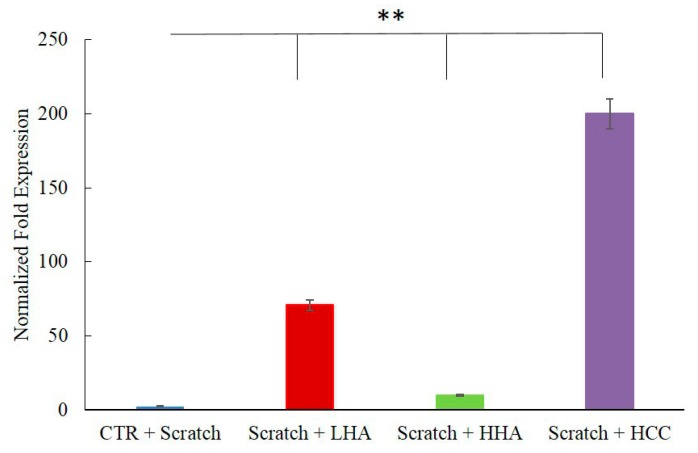
Effects of LHA, HHA, and HCC on HBD-2 expression in HaCaT/HDF co-cultures in comparison with a control sample. HaCaT/HDF cells were scratched and treated with HA and its complex. At 24 h, the total RNA was extracted and qRT-PCR was performed to determine the gene expression of HBD-2. **p < 0.01 for HCC, HHA, and LHA vs. CTR.

During the wound-healing process, infection by pathogens is frequently the major issue hampering repair. Therefore, we evaluated HBD-2 expression in scratched co-cultures. As the expression of this antimicrobial peptide is naturally up-regulated by IL-1β, it was more reliable to evaluate its modulation in the absence of an inflammatory stimulus. Figure 4 shows the significant increase in HBD-2 in the presence of LHA and HCC (35-fold and 100-fold, respectively).

#### 2.2.3. Inflammation and Wound Closure

**Figure 5:**
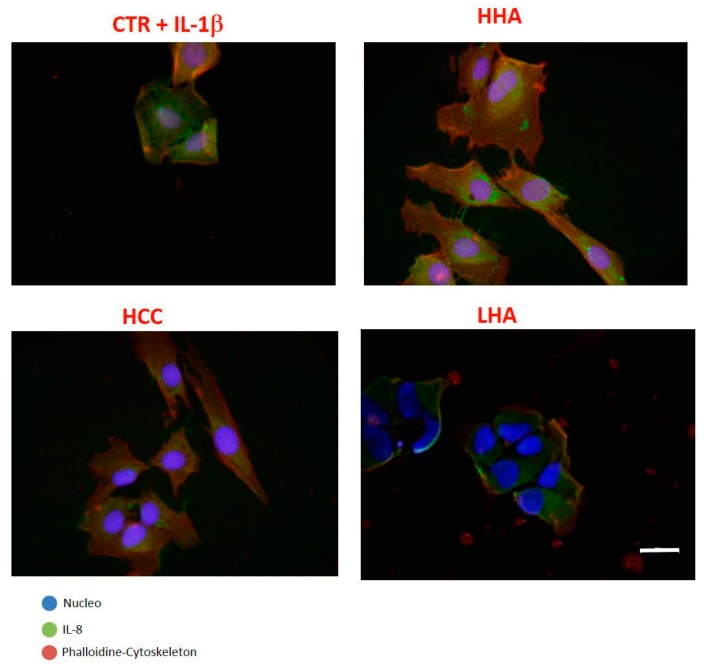
Expression of IL-8 in HaCat/HDF co-culture. Cells were stimulated with IL-1β for 2 h and then treated with HHA, LHA, and HCC at 0.4% *w*/*w*, as well as a control for comparison. Images were taken 24 h after treatment. The panels show triple immunofluorescence analysis for cytoskeleton (red), nucleus (blue), and interleukin (green). In the presence of HCCs, the expression of IL-8 decreased. Scale bar represents 100 μm.

In the presence of IL-1β, immunofluorescence demonstrated that IL-8 expression was reduced for HHA-treated, and especially HCC-treated, cells (Figure 5). These results further support the anti-inflammatory role of HCC.

**Figure 6:**
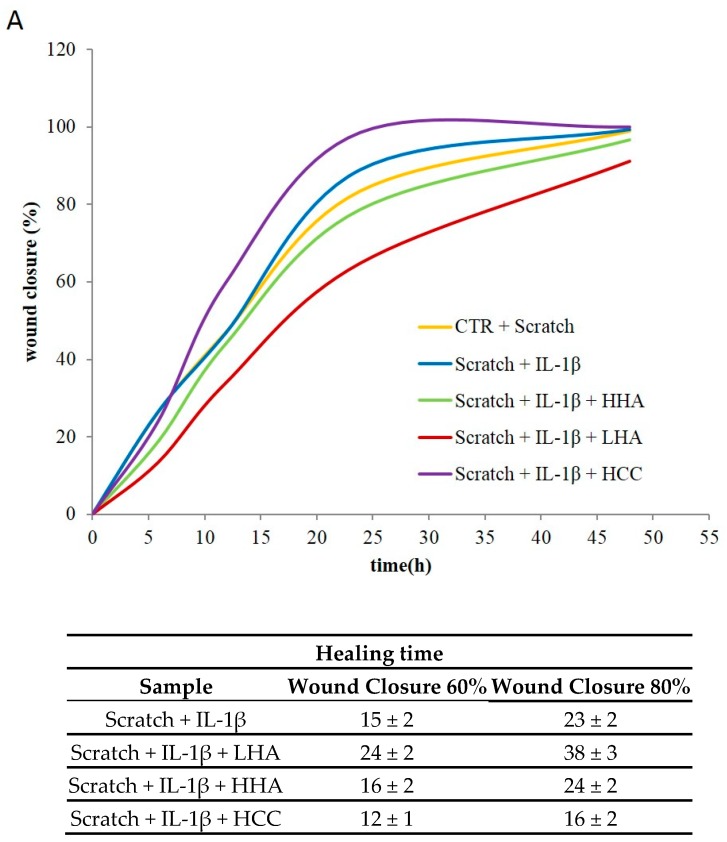
Effects of HHA, LHA, and HCC on HaCaT/HDF scratch test co-cultures. (**A**) Repair area percentage for the control and in the presence of treatments. The repair area percentage is given as ((*A_0_* – *A_t_*)/*A_0_*) × 100, where *A_0_* and *A_t_* are the wound areas just after scratching and after a time *t* of healing, respectively. The addition of IL-1β had no effect on wound repair. (**B**) The healing time for each treatment to achieve 60% and 80% wound closure.

For the scratch test in the presence of IL-1β, the percentage of wound closure is plotted in Figure 6 comparing the relative cell migration during the wound healing process in the control condition and in the presence of HA treatments. We observed that the natural wound closure was not conditioned by IL-1β treatment, probably due to the presence of fibroblasts. It is evident that the scratch closure occurred at a faster rate in the presence of HCC. After 15 h of culture, less than 60 ± 2% of the scratched area was repaired, whereas in the presence of HCC, it had already reached (75–85) ± 3%. Total wound closure occurred at 20 ± 2 h in the presence of HCC, whereas more than 40 h were needed for all other samples.

The statistical analysis was run on five measurements for each of the experimental conditions at specific wound repair areas (60% and 80%) as reported in Figure 6B. Specifically, LHA was slower than CTR in the repairing process, HHA was not significantly different from the IL-1β treated CTR, while HCC proved significantly faster than all the others (*p* < 0.05).

**Figure 7:**
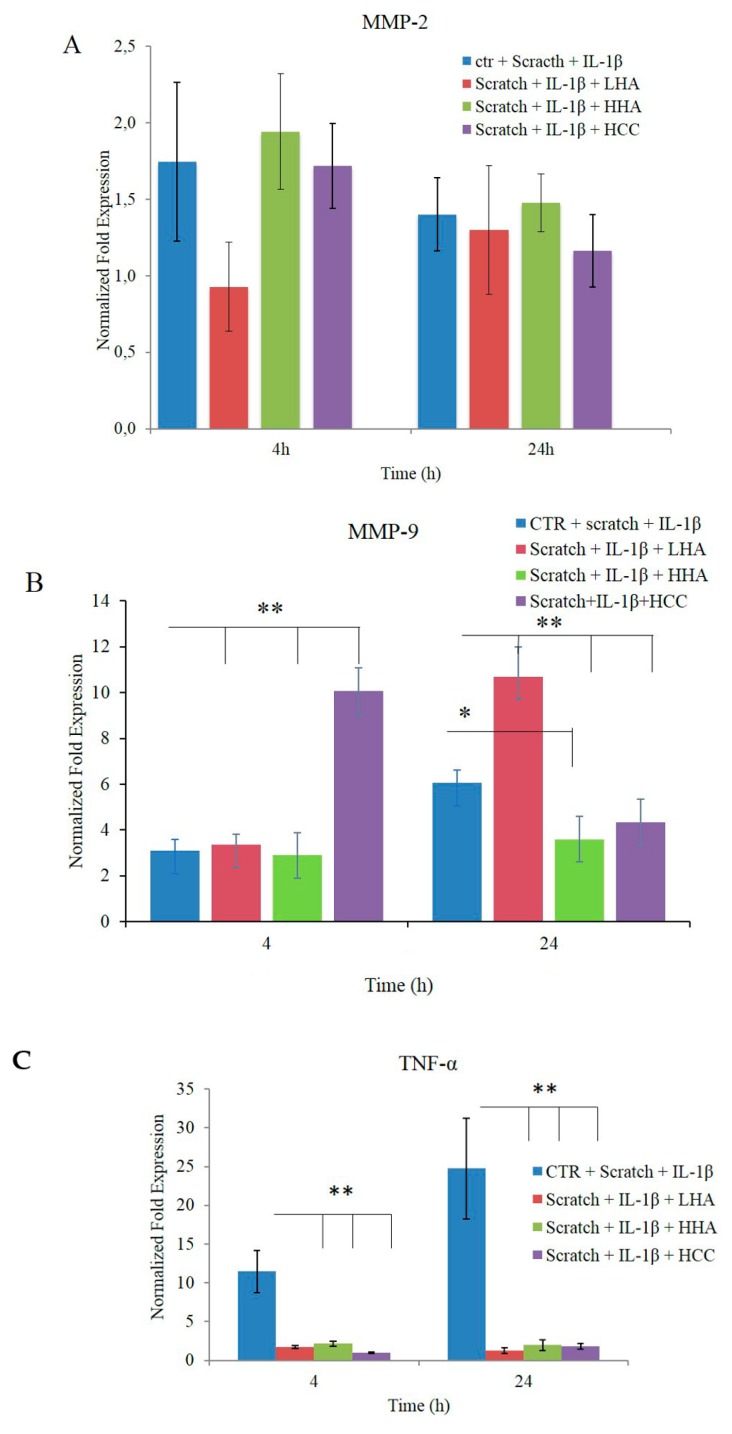

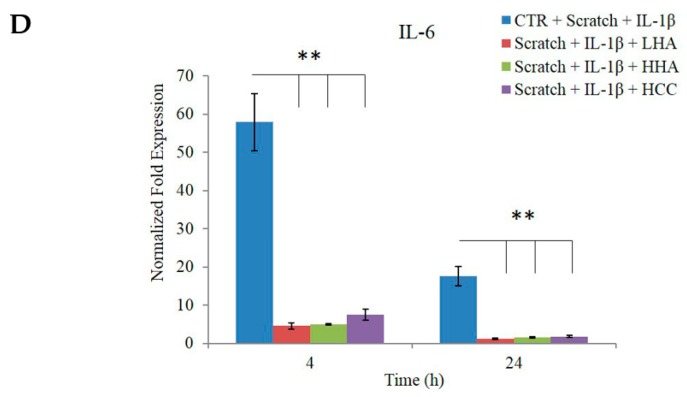
Effects of LHA, HHA, and HCC on mRNA expression in HaCaT/HDF co-cultures in comparison with a control sample. The HaCaT/HDF cells were scratched and stimulated with IL-1β for 2 h in DMEM with 10% FBS, and then treated with HA and its complex. Data are presented as mean ± SD. At 4 and 24 h, the total RNA was extracted and qRT-PCR was performed to determine the gene expression of (**A**) MMP-2, (**B**) MMP-9, (**C**) TNF-α, and (**D**) IL-6. *p < 0.01 for LHA, HHA, and HCC vs. control (CTR) at 4 h or LHA and HCC vs. CTR at 24 h; **p < 0.05 for HHA vs. CTR at 24 h.

Concerning specific remodeling biomarkers, we found that IL-1β treatment alone already up-regulated MMP-2 expression by 1.5–2-fold at 4 h, and all the treatments, except for LHA, were in agreement with recently reported results [25]. At 24 h, all the treatments, except for LHA, presented a reduction of MMP-2 expression coherent with the accomplished wound closure. However, MMP-2 for LHA treatments was higher at 24 h than the one found at 4 h. In fact, LHA treatment allowed for only 60–70% closure in 24 h (Figure 7A).

Up-regulation of MMP-9 occurred in the presence of HCC in a short time frame (4 h after treatment). At 24 h, the expression of MMP-9 decreased with HCC or HHA treatment compared to the control and LHA samples, confirming the faster wound closure in the presence of the first two treatments (Figure 7B).

Moreover, TNF-α and IL-6 gene expression confirmed the anti-inflammatory effect of HA (Figure 7C,D), as previously reported for a single culture [16,21].

#### 2.2.4. Bio-Plex Assay

**Figure 8:**
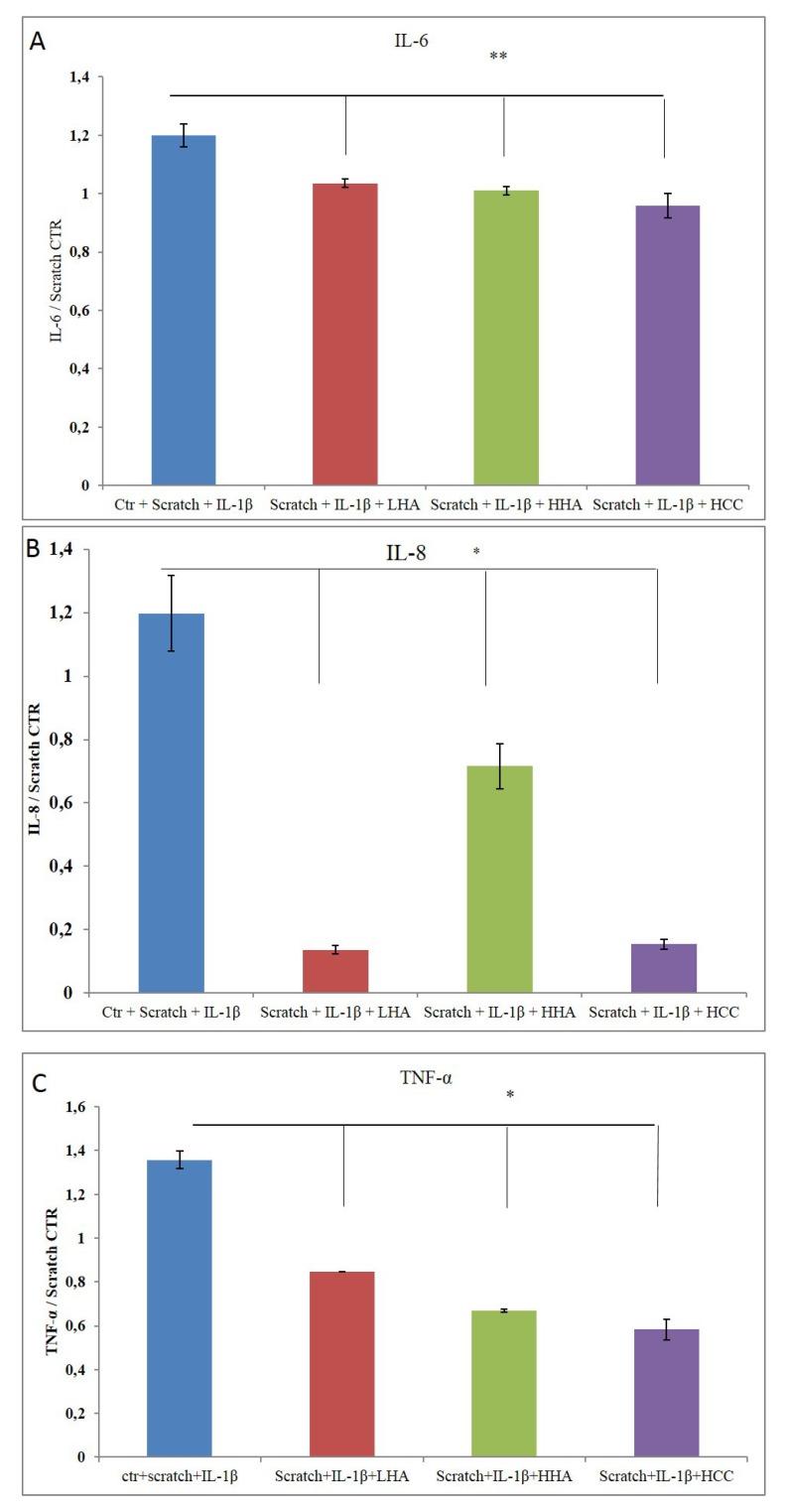
Quantification of cytokines. (**A**) TNF-α, (**B**) IL-6, and (**C**) IL-8 were evaluated using Bio-plex. The data were normalized with respect to the scratch control (CTR) and reported as ratios of protein concentration (pg/mL) as mean ± SD. *p < 0.01 for HCC, HHA, and LHA vs. CTR in (**A**); *p* < 0.05 for HCC vs. CTR in (**B**); or *p* < 0.01 for HCC, HHA, and LHA vs. CTR in (**C**).

Of all the cytokines analyzed using the Bio-plex assay [Granulocyte-Macrophage Colony-Stimulating Factor (GM-CSF), interferon γ (INF-γ), IL-2, IL-4, IL-6, IL-8, and TNF-α)], only IL-6, IL-8, and TNF-α were modulated by our treatments. Figure 8 reveals the appreciable reduction in TNF-α and IL-8 protein expression, confirming a positive effect of HA-based formulations, specifically HCCs, on both biomarkers. The results show that there was a remarkable reduction in protein levels. IL-6 exhibited a slight reduction (20–25%) in the presence of all HA-based creams (Figure 8B).

## 3. Discussion

HA plays a pivotal role in wound healing through different mechanisms. In this experimental work, we investigated how the contemporary presence of different sizes of HA could eventually improve HA-based medical devices for wound care. The use of HCCs based on HHA (≈1200 kDa) and LHA (≈100 kDa) chains obtained through NaHyCo® technology was previously tested and demonstrated tissue repair and human adipose stem cell differentiation possibly involved in tissue remodeling [15,26,27,28,29,30].

Commercialized ointments are already available based on medium–low-molecular-weight HA (200–300 kDa) and have been shown to promote wound healing thanks to their peculiar characteristics of increasing the migration of cells through the dermal layer while reducing inflammation [16]. At the same time, high molecular weights have been widely exploited, even if with controversial outcomes and recommendations regarding wound healing treatment [30]. More recently, the commonly used LHA (200–300 kDa) was complexed (interpenetrated) with thicker, more viscous, and adhesive HHA macromolecules, resulting in a positive effect on the wound healing process and biorevitalization of the skin [31,32]. Hydrodynamic and rheological characterizations of novel HCC formulations are performed to ensure that the viscosity of novel potential creams is suitable for specific treatments and will not hamper natural cell migration. Specifically, the lower viscosity of HCCs compared to the HHA-based samples may improve spreadability. However, compared to the lower viscosity of the LHA sample, HCC may be retained longer on the treated area, suggesting a potential long-lasting efficacy. Moreover, HCCs, with respect to HHA alone, also preserve the degradation of HA, at least regarding hyaluronidase hydrolysis [15], ensuring protracted persistence of the product on the application site.

In the framework of this research, we aimed at reproducing a skin-stressful condition with a double insult. Preliminary experiments confirmed an increase in elastin production in the HaCaT/HDF model when the cells were treated with the HCC formulation, proving its positive effect on skin restoration. These results agree with previous studies, notwithstanding the use of different HA sizes and higher concentrations with respect to the samples used previously [27,28,32,33].

In the present study, a new in vitro model of wound healing based on a HaCaT/HDF monolayer culture was used to simulate the natural skin repairing conditions as much as possible. In addition, the presence of an inflammation agent (i.e., IL-1β) prompted an increase in cytokines at the gene and protein level, as expected, confirming a higher inflammation status with respect to scratching alone. However, this higher inflammation status did not correspond with a lower wound-healing rate. When only the HaCaT monolayer was scratched and subjected to treatment with IL-1β, the repair rate slowed with respect to scratching alone (data not shown). This different behavior is probably due to the presence of fibroblasts that, when treated with an inflammatory agent, lead to increased MMP levels, prompting matrix remodeling and the repair process itself [33].

In the present study, we found that IL-1β treatment alone already up-regulated MMP-2 expression at an early stage; this might be coherent with a recently reported experiment that used laser as insult and gained an increase in MMP-2 expression that was 30% higher with a first treatment and 50% higher with a second treatment [25]. In addition, we found that MMP-9 expression was higher at the beginning for an HCC treatment with respect to the others. In fact, there was an opposite modulation trend. This was probably due to the higher reparation rate found for HCC-treated cells. In addition, the reduction in inflammatory biomarkers found at the transcriptional and protein levels (TNF-α, IL-6, and IL-8) indicates that the cells neutralized the stressful conditions thanks to the hyaluronan effect. These data are in agreement with recent literature [15,34] showing the key role of HA and its fragments in regulating inflammation-associated genes by recruiting inflammatory cells, providing protection against tissue damage. These outcomes confirm the broad anti-inflammatory action of glycosaminoglycans in accordance with the results obtained using other cellular models (e.g., chondrocytes) [34]. In addition, our data suggest that HCC treatment induced an increase in the expression of HBD-2, an inducible antimicrobial peptide linked to the innate immune response. Thus, while the modulation of proinflammatory responses protected the host from the detrimental effects of overwhelming inflammation, the antimicrobial peptide HBD-2 was up-regulated, suggesting continuous host protection against infection risk. HBD-2 may promote adaptive immune responses by recruiting dendritic and T cells to the site of a microbial invasion [34]. Moreover, it can stimulate cell migration and proliferation [35], and to be a modulator of angiogenesis [36]. These findings support the idea that HBD-2 induction may improve wound healing and restrict bacterial overgrowth even in vivo, moving toward the restoration of physiological conditions.

In conclusion, we can assert that HCCs of hyaluronic acid may behave differently than other forms of linear HA used to date. In this case, the results support HCCs as an innovative key ingredient in topical applications, as it was a consistent viscoelastic gel that could be applied as a cream on wounds to improve the structure of the newly formed tissue and accelerate the repair process.

## 4. Materials and Methods

### 4.1. Materials

HHA (sodium hyaluronate, Altergon topical use; code: 396253002, batch no: 1000004608) and LHA (sodium hyaluronate for medical device topical use, Altergon; code: 2484002, batch no: md16003) were generously gifted by Altergon s.r.l. (Morra De Sanctis (AV), Italy).

HaCaT cells (Istituto Zooprofilattico, Brescia, Italy), a spontaneously transformed non-tumorigenic human keratinocyte cell line, were cultured in Dulbecco’s modified Eagle’s medium (DMEM) supplemented with 10% (*v*/*v*) heat-inactivated fetal bovine serum (FBS), 100 U/mL penicillin, and 100 µg/mL streptomycin. A human dermal fibroblast cell line immortalized with hTERT (HDF cells, BJ-5ta, and ATCC CRL-4001) was cultured in a 4:1 mixture of DMEM and Medium199 supplemented with 0.01 mg/mL hygromycin B and 10% (*v*/*v*) FBS (ThermoFisher Scientific, Rome, Italy). All materials for the HDF culture were purchased from ATCC (Manassas, VA, USA). The cells were grown on tissue culture plates (BD Falcon, Milan, Italy) in an incubator with a humidified atmosphere (95% air/5% CO_2_ *v*/*v*) at 37 °C. All materials and reagents for HaCaT culture were purchased from Flow Laboratories (Milan, Italy). The cells were grown on tissue culture plates (Corning Incorporated, New York, NY, USA) in an incubator with a humidified atmosphere (95% air/5% CO_2_ *v*/*v*) at 37 °C. Collagen was purchased from Sigma-Aldrich (Milan, Italy).

### 4.2. Sample Preparation

HCCs were prepared by dissolving an equal amount (ratio 1:1) of HLA and LHA in a phosphate buffer solution overnight under gentle shaking, achieving a final concentration of 32 g/L. The solubilized formulation was then treated with the patented thermal process NaHyCo® (https://www.ibsagroup.com/it/innovation/technologies.html). The formulation was diluted to 0.4% and 0.2% (*w*/*w*) in a physiological buffer or directly in DMEM (growth medium), respectively, before using it for the experiments.

### 4.3. Hydrodynamic Characterization and Rheological Measurements

Chromatographic analyses of samples were performed using size exclusion chromatography coupled with a triple detection array (SEC–TDA) equipment from Viscotek (Malvern Instruments, Cambridge, U.K.). A detailed description of the system and its analytical conditions are reported elsewhere [2,23]. Rheological measurements were carried out using a Physica MCR301 oscillatory rheometer (Anton Paar, Ostfildern-Scharnhausen, Germany) equipped with a DG26.7/T200/AL (double gap) and a Peltier temperature control. Flow curves (dynamic viscosity as a function of the shear rate) were recorded as reported elsewhere [2]. Measurements were carried out at 25 °C over a shear rate of 0.01 to 300 s^−1^. From each flow curve, we derived the zero-shear viscosity (η_0_, dynamic viscosity in the Newtonian plateau).

### 4.4. In Vitro Scratch Test

Wound healing was evaluated using an in vitro scratch assay performed on cellular monolayers and monitored using time lapse video microscopy for 48–72 h (TLVM, Okolab, Pozzuoli, Napoli, Italy). Alternatively, after the scratch and prior to the addition of the HA formulations, treatment with 50 ng/mL IL-1β was performed for 2 h as an additional injury to cause inflammation. Time-lapse experiments were reported previously [15,21,22]. Briefly, to test the effect of HA on the rate of wound closure, the scratched monolayers were incubated with 0.4% *w*/*v* HHA, LHA, and HCC in DMEM with 1% FBS. Fresh serum-supplemented medium (1% *v*/*v* FBS) was used as a control. The station allowed us to monitor temperature (37 °C) and CO_2_ (5% in air) through a touch-screen interface and remote-controlled motor that permitted micrometer movements and repositioning of the stage incubator along the x-, y-, and z-axes cyclically over time. The images of the “wound closure” phenomenon captured using a CCD camera were processed with software (OKO-Vision 4.3, http://www.oko-lab.com/) that allowed us to quantitatively analyse the wound healing by displaying the recorded images.

Wound closure was examined by monitoring the cell covered area using an automatized software that allowed us to measure the area at time zero (t_0_) and the area for each image acquired. The developed software calculated the wound closure exploiting the following equation:

$$\;\left(\frac{A_{0}-A_{t}}{A_{0}}\right)\times 100$$

where *A_0_* and *A_t_* are the wound areas just after scratching and after a time *t* of healing, respectively. Each scratch assay was performed in duplicate.

### 4.5. Quantitative RT-PCR

The expression of ELS, MMP-2, MMP-9, HBD-2, TNF-α, and IL-6 mRNA was evaluated using quantitative real-time PCR (qRT-PCR). A full description of the gene expression was reported by D’Agostino et al. [22]. Briefly, total RNA was extracted from cells using TRIzol^®^ (Invitrogen, Milan, Italy). A total of 1 µg of DNase-digested total RNA (DNA-free kit; Ambion-Applied Biosystems, Foster City, California, USA) was reverse-transcribed to cDNA using the Reverse Transcription System Kit (Promega, Milan, Italy). The iQ^TM^ SYBR® Green Supermix was used (Bio-Rad Laboratories s.r.l., Milan, Italy) to analyze gene expression using appropriate primer pairs. All reactions were performed in triplicate, and the relative expression of specific mRNA was determined by normalizing it to the housekeeping gene hypoxanthine guanine phosphoribosyl transferase (*HPRT*). The fold-change in gene expression was calculated using the comparative threshold method (ΔΔC*t* = difference in ΔCt between HA-treated cells and control) and the results are expressed as the normalized fold expression relative to controls using Bio-Rad iQ™5 software I cycler version 4006 (Bio-Rad Laboratories s.r.l, Segrate (MI) - Italy) [24].

### 4.6. Western Blotting

Proteins were extracted after 4 h and 24 h using a RIPA lysis buffer and the concentrations were determined using Bio-Rad protein assay reagent (Bio-Rad Laboratories s.r.l.). Equal amounts of protein (40 µg) were loaded onto a polyacrylamide gel for SDS-PAGE and transferred to a nitrocellulose membrane. The filters were incubated overnight at 4 °C with antibodies against elastin (1:250), tubulin (1:500), and actin (1:1000). The membranes were washed three times for 10 min and incubated with anti-rabbit (1:10,000), anti-mouse (1:5000), or anti-goat (1:5000) horseradish peroxidase-conjugated antibodies (Santa Cruz Biotechnology, Dallas, TX, USA) for 1 h. Protein levels were normalized with respect to the signal of anti-actin polyclonal antibody. Blots were developed using the ECL system (Enhanced Chemiluminescence) (Amersham Biosciences, Little Chalfont Buckinghamshire United Kingdom) according to the manufacturer’s protocol. Densitometric analyses were performed using a Gel Doc 2000 UV System and the Gel Doc EZ Imager (Quantity One software, System ID 4020DABD, Bio-Rad Laboratories s.r.l.).

### 4.7. Immunofluorescent Staining

The expression of elastin after 24 h of HA treatment and IL-8 on HaCaT/HDF co-cultures pre-treated with IL-1β before HA formulations addition were evaluated in immunofluorescence experiments. Cell co-cultures were stained simultaneously to ensure uniform conditions for fluorescence analysis. Cells were fixed in 4% PFA (paraformaldehyde) for 15 min, rinsed with PBS buffer, and blocked by blocking solution (5% normal serum and 0.3% Triton X-100 in PBS) for 60 min at room temperature. The cell co-cultures were then incubated overnight at 4 °C with primary antibodies against elastin (monoclonal mouse antibody, 1:50; Santa Cruz Biotechnology) and IL-8 (monoclonal rabbit antibody, 1:50; Santa Cruz Biotechnology). After washing with PBS three times, glass microscope slides were incubated with Alexa Fluor® 488-conjugated goat anti-rabbit IgG (H+L) secondary antibody (1:100; Life Technologies, Monza, Italy) or Alexa Fluor® 568-conjugated goat anti-mouse IgG (H+L) secondary antibody (1:100; Life Technologies). For visualization of actin filaments, cells were stained with 50 mg/mL fluorescent phalloidin TRITC (Tetramethylrhodamine) conjugate (Sigma-Aldrich) solution in PBS for 40 min at room temperature. Nuclei were counterstained using Hoechst for 10 min (0.5 µg/mL Sigma-Aldrich). Coverslip slides were obtained using ProLong™ antifade mountant (Life Technologies). Samples were then examined under the Nikon fluorescence microscope and analyzed by Nikon (Multicolor Package, Leica Wetzlar, German).

### 4.8. Cytokine Quantification Using Bio-Plex

The levels of the following cytokines were quantified using Bio-plex (Human Cytokine Panel 8-plex; Bio-plex, Bio-Rad Laboratories s.r.l.) assays with GM-CSF, INF-γ, IL-2, IL-4, IL-6, IL-8, and TNF-α (pg/mL) according to the manufacturer’s instructions. Briefly, supernatants (50 μL) were incubated overnight with antibody-coated beads under mild agitation at 4 °C. Detection antibodies were incubated for 1 h at room temperature, and the fluorescent conjugate streptavidin-phycoerythrin was added to each well and incubated for 30 min at room temperature. Cytokine levels (pg/mL) were analyzed using a Bio-Plex array reader (Luminex, Austin, TX, USA). The analytical concentrations were calculated using a standard curve according to the manufacturer’s instructions [20].

**Figure 9:**
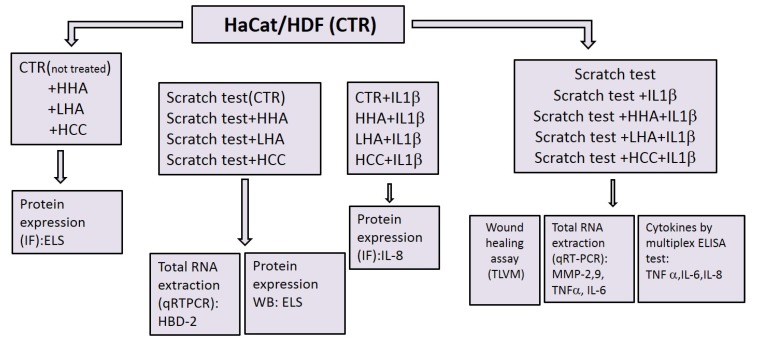
Schematic diagram that shows the experiment set up. (ImmunoFluorescence (IF), Western Blotting (WB), Elastin (ELS), Enzyme-Linked Immunosorbent Assay (ELISA), Time Lapse Videomicroscopy (TLVM), quantitative Real Time Polymerase Chain Reaction (qRT-PCR).

A schematic diagram of the experiments is given in Figure 9.

## 5. Conclusions

In vitro cell co-culture models subjected to stressful conditions to mimic injuries, when supported by TLVM and biomolecular fingerprints, allowed for contemporary evaluation of different products and molecules in highly standardized conditions to develop novel medical devices or pharmaceutical formulations. The promising results obtained in this study regarding increased scratch closure rates and positive the modulation of remodeling biomarkers, together with reduced inflammation, suggest that HCCs could be an innovative and reliable tool in wound management.

## Figures and Tables

**Table 1:** Zero-shear viscosity of HHA, LHA, and HCC at 0.4% *w*/*v* in PBS after thermal treatment.

Samples Post Thermal Treatments	Zero-Shear Viscosity (η_0_) (mPa⋅s)
HHA	94.73 ± 0.46
LHA	3.95 ± 0.01
HCC	16.60 ± 0.06

HHA, high and low molecular weight; LHA, low molecular weight; HCC, hybrid cooperative complex.
